# New Method of Rectal Nutrition

**Published:** 1874-02

**Authors:** 


Nutritious Injection—New Method of Nourishing Patients.—M. Leube has had the idea of rendering the digestion in the large intestine very active by carrying into this organ, simultaneously, digestible substances and a substance which is digestive. The pancreas of the hog constitutes the latter. The mass to be digested is made in the following manner: Fifty to one hundred grammes of the pancreas of the hog or ox, carefully deprived of its fatty tissue, are chopped fine and mixed with one hundred and fifty to two hundred grammes of beef. The two substances are pounded in a mortar with warm water, a magma being formed which is injected by means of a syringe, the nozzle of which is of large calibre. The injections thus composed have given good results with dogs. A fecal mass is formed after the injections which is quite analogous to the ordinary matters; the fat and the albumen are digested by the large intestine. This method of nutrition has been applied by the author to two patients. In one case there was a cancer of the upper portion of the digestive tube, in the other the patient could not receive any aliment without rejecting it by vomiting. In these cases the pancreatic substances did not cause any diarrhœa; they remained in the intestine for from twelve to thirty-six hours without producing any evacuations, and the patients did not experience any pain. After the injections the stools became more full; but at first the injections were not entirely retained, the patients rejecting a part of the injected mass undigested. This *melange* is, according to the author, superior to all the substances which are recommended for nourishing by the large intestines.—*Gazette Hebdomadaire.*

